# Effects of COVID-19 pandemic on household food waste behaviour in Iran

**DOI:** 10.1016/j.heliyon.2022.e11337

**Published:** 2022-10-29

**Authors:** Mohammad Sadegh Allahyari, Soroush Marzban, Hamid El Bilali, Tarek Ben Hassen

**Affiliations:** a Department of Agricultural Management, Rasht Branch, Islamic Azad University, Rasht, Iran; b North-West University, Faculty of Economic and Management Sciences, South Africa; c Department of Agricultural Extension and Education, School of Agriculture, Shiraz University, Iran; d International Centre for Advanced Mediterranean Agronomic Studies (CIHEAM-Bari), Via Ceglie 9, Valenzano (Bari), Italy; e Program of Policy, Planning, and Development, Department of International Affairs, College of Arts and Sciences, Qatar University, P.O. Box: 2713, Doha, Qatar

**Keywords:** COVID-19, Food behaviour, Food consumption, Food shopping, Food waste, Iran, Near east and North Africa

## Abstract

This research aims to determine the influence of COVID-19 on consumer knowledge, attitudes, and behaviours related to food waste in Iran. From April 24 to May 24, 2020, an online survey was conducted in Iran with a standard questionnaire delivered in Persian. Descriptive statistics and various non-parametric tests were used to analyse the survey results. The results reveal significant changes in how consumers shop and interact with food, with implications on household food wastage. Indeed, according to the survey findings: (i) Iran's households have a positive attitude toward reducing food waste; (ii) food waste dropped during the pandemic; (iii) consumers made fewer shopping trips and spent less on groceries during the pandemic; (iv) food waste did not increase during the month of Ramadan. The survey results provide valuable insights to reduce food wastage and address food security risks during the COVID-19 pandemic in Iran. The paper results contribute to a better understanding of food waste management behaviours and the influence of the COVID-19 pandemic in Iran, which is paramount to designing effective, efficient, and sustainable recovery plans and policies.

## 1 Introduction

COVID-19 triggered a devastating global socio-economic crisis, with severe disruptive impacts on agri-food systems and food consumption (El Bilali et al., 2021b; FAO, 2020; Galanakis, 2020; HLPE, 2020; iPES Food, 2020; One Planet Network, 2020; UNSCN, 2020). Strong containment measures, such as home confinements, social distancing, and lockdowns, enforced by numerous governments throughout the globe to stop the virus, have disturbed the relationships between the various components of the food system from farmers to processors to consumers, resulting in an imminent worldwide food emergency (Galanakis, 2020; United Nations, 2020). Further, the COVID-19 pandemic significantly influenced people's daily lives, including various substantial implications on food management and consumption behaviours, such as food waste (Jribi et al., 2020; Rodgers et al., 2021a). Furthermore, almost two years after the Coronavirus was identified, the pandemic is far from over, with several countries still dealing with substantial infections. Even those in control of the virus are anxious about upcoming waves, especially with the new and more infectious variants, such as Delta and Omicron (WHO, 2021). The threat of the new infections and waves may result in more lockdowns or the maintenance of current severe restrictions in the coming months, causing further disruption to global food systems.

Nonetheless, the pandemic's consequences on food waste remain perplexing. On the one hand, widespread panic-buying and stockpiling during the onset of the pandemic led to a rise in household food waste, particularly for fresh foods, owing to storage issues, poor cooking practices, or overcooking (Ben Hassen et al., 2021d; Berjan et al., 2021; Cranfield, 2020; Jribi et al., 2020; Lahath et al., 2021). On the other hand, most consumers in many countries improve their food management behaviours, including more extensive food pre-planning (such as making a shopping list), improved in-home food storage, and less-conventional cooking and preparation methods (such as using leftovers), resulting in less food waste (Ben Hassen et al., 2021a; Principato et al., 2020; Renzo et al., 2020). Adopting these practices is driven by various motives, including the desire to avoid going to the grocery store due to the perceived virus risk, save money, and be less rushed for time. However, the socio-economic crisis caused by the COVID-19 pandemic (i.e. food availability, limited mobility, and income loss) is more likely to impact consumers' behavioural shifts toward minimising food waste than a pro-environmental purpose (Jribi et al., 2020). Indeed, COVID-19 caused a worldwide economic and financial crisis, rising unemployment rates, and global poverty (International Monetary Fund, 2020). It seems that out of necessity, consumers decreased their food waste. During a crisis, consumers are more inclined to save rather than waste, resulting in a considerable drop in waste generation (Durante and Laran, 2016), as shown in prior recessions in Greece and Italy (Fanelli and Florio, 2016; Martinengo, 2014). Nonetheless, the effects of COVID-19 vary per country, depending on epidemiological factors as well as socio-economic development. Iran, a middle-income country subject to heavy American sanctions that have caused significant socio-economic issues, is particularly an interesting case study in this regard.

On February 19, 2020, Iran confirmed the first case and the first death of COVID-19 in the city of Qom (140 km south of Tehran, the capital) (Aljazeera, 2020). As of February 16, 2022, with 6,835,221 confirmed cases and 133,886 total deaths, Iran is the most affected country in the NENA (Near East and North Africa) region and one of the most impacted worldwide (WHO, 2022). The Iranian government has made many efforts to prevent the propagation of COVID-19, including the closure of businesses and educational institutions as well as curfew and lockdown (Arab News, 2020; BBC, 2020). These restrictions were progressively relaxed beginning in April 2020. However, infections and fatalities have grown periodically with successive waves, leading the government to impose certain restrictions. Recently, Iran has seen an alarming spike in the number of COVID-19 infections, fueled by the spread of the Omicron variant across the country. As a result, the government has warned that certain restrictions may be reinstated (Al Jazeera, 2022; Iran International, 2022).

These efforts were crucial in containing the pandemic. However, they may have caused disruptions in daily life and altered food consumption habits in Iran. In general, data about household food wastage in Iran are scarce and fragmented. Moreover, most studies focused on specific locations or cities (Alavi Moghadam et al., 2006; Fami et al., 2019), or food loss (e.g. during agriculture production, harvesting, etc.) rather than on food waste (Asadi et al., 2010; Fallah and Rasouliazar, 2015; Omidi et al., 2014). Additional data and information is required in Iran for COVID-19 to be evaluated in terms of its effects on food consumption and waste. Accordingly, this research aims to determine the influence of COVID-19 on consumer knowledge, attitudes, and behaviours related to food waste in Iran. Furthermore, the fasting month of Ramadan is examined to see whether it influences Iranian family waste behaviours. It would be, to our knowledge, the first study of its kind in Iran. Before we expose the results and discuss them (Section 4), we present a literature review (Section 2) and then our methodology (Section 3).

## 2 Materials and methods

### 2.1 Sampling methods

The research was based on an online survey administered in Iran using a structured questionnaire. Data was collected using Porsline (https://survey.porsline.ir), an online survey tool. The study is aimed at Iran's general adult population (people over 18). It used the snowball sampling method. We used a non-probability sampling technique because survey participants were chosen randomly and voluntarily. With the COVID-19 pandemic restrictions, snowball sampling provides significant advantages, and other sampling strategies are unlikely to succeed.

The snowball sampling approach was developed following Noy (2008) and Heckathorn (2011), who proposed a multiple referral approach. This method benefits from not being readily interrupted or halted and minimises possible sample bias (Hermsdorf et al., 2017). In addition to email and social media (WhatsApp and Telegram), the poll was distributed through several institutional communication channels. Due to their widespread usage in Iran, these two social media platforms were selected (33 million users out of a total population of 82.8 million) (Tehran Times, 2019).

Furthermore, the research was carried out per the Helsinki Declaration principles. All methods involving research participants were approved by the Islamic Azad University Human Subjects Institutional Review Board (HSIRB). The study's participation was entirely voluntary. Each participant was informed about the study's goal and context before signing informed consent regarding information sharing and privacy regulations. There was no compensation for participation.

### 2.2 Data collection and questionnaire design

The questionnaire was developed and adapted based on previous studies on the impact of the COVID-19 pandemic on food-related activities, including food waste performed in the MENA (Middle East North Africa) region (Ben Hassen et al., 2021e; Ben Hassen et al., 2022a, Ben Hassen et al., 2022b; El Bilali et al., 2021a), and the Balkans (Ben Hassen et al., 2021b, Ben Hassen et al., 2021c; Berjan et al., 2021). For the Iranian context, the questionnaire was translated into Persian. Conducted from April 24 to May 24, 2020, the survey was sent to 1746 persons, and 774 questionnaires were completed (response rate of 62%) and used for the analysis. The questionnaire was made up of 32 one-option and multiple-choice questions divided into nine sections: (1) Socio-demographics; (2) Food shopping behaviour and estimate of food expenditures; (3) Knowledge of food labelling information; (4) Attitudes towards food waste; (5) Extent of household food waste; (6) Economic value of household food waste; (7) Willingness and information need to reduce food waste; (8) Food waste and purchasing habits during the COVID-19 first wave; and (9) Food waste and purchasing habits during Ramadan (Appendix A).

The questionnaire was carefully designed to guarantee the quality of the survey data, minimise the risk of common method variation and reduce the probability of respondents misinterpreting the questions. In addition, some precautionary steps were put in place. Before it was made available, the survey was double-checked. Before conducting the survey, an expert panel did a qualitative content validity examination. Based on experts’ opinions, irrelevant elements were deleted, and the remaining items were updated to be more precise and clear. Second, 23 persons participated in a pilot study of the questionnaire. This process ensured that responders could understand and correctly answer the questions. Based on the results of the pretests, we made adjustments to the questionnaire and sent it out. Self-reporting is used in the research.

### 2.3 Data analysis

Our data analysis is based on various methods: descriptive statistics, chi-square tests of independence, Wilcoxon statistical test, and cluster analysis. SPSS was used to analyse primary survey data, including descriptive statistics (frequency counts and percentages). The correlation with demographic variables was tested using Chi-square tests of independence. Also, the Wilcoxon test was used to distinguish the differences between shopping behaviour and food waste during Ramadan and COVID-19 lockdown. Further, cluster analysis was used. Hierarchical cluster analysis (HCA) and K-means cluster analysis are the two main clustering algorithms (KCA) types. As a more theoretical approach, hierarchical cluster analysis is often used in conjunction with K-means cluster analysis (Gough, 2001).

In this research, we used the K-means cluster analysis technique, one of the most prominent clustering techniques, to produce representative groupings for the examined data and classify variables such as shopping duration, shopping place, etc. It was used successfully in several studies on food waste in households in many countries, including the United Kingdom (Mallinson et al., 2016), Germany (Richter, 2017), Italy (Aureli et al., 2021) and Romania (Pocol et al., 2020). Furthermore, this technique was employed in various studies on the impact of the COVID-19 pandemic on food waste (Amicarelli et al., 2021a, Amicarelli et al., 2021b; Yetkin Özbük et al., 2021).

This research has no hierarchical clusters; each cluster has at least one item, with no overlaps. It is the goal of K-means clustering to divide an *n* observation (x_1_, x_2_,…, x_n_) into k (≤n) sets, i.e., *S* = {S_1_, S_2_,…, S_k_}, in order to minimise the sum of squares within each subset. Using K-means clustering, it is possible to determine the proper cluster for all household types and accurately describe the group (Marzban et al., 2016).

## 3 Results

### 3.1 Socio-demographic characteristics of the respondents

According to Table 1, most respondents were in their forties and fifties (33.6% were 35–45 years old). Gender equality was nearly achieved (53% of the respondents were women, and 47% were men). Furthermore, 60.2% were married with children, 42.2% were professionally active, and 98.9% were highly educated (with a diploma and above). This was due to method of administration bias, as the survey was primarily distributed through the internet and social media, which young and educated people primarily used.

**Table 1:** Sociodemographic characteristics of the participants (n = 773).

Variable	Item	Frequency	% of respondents
**Age (years old)**	Less than 25	92	11.9
	25 to 35	187	24.2
	35 to 45	260	33.6
	45 to 55	170	22.0
	55 and more	64	8.3
**Gender**	Female	410	53.0
	Male	363	47.0
**Education Level**	Primary	2	0.3
	Secondary	6	0.8
	Diploma	88	11.4
	Master degree	273	35.3
	Higher Education (e.g. PhD)	404	52.3
**Occupation**	In paid work (full-time or part-time)	326	42.2
	Student	66	8.5
	Unemployed	28	3.6
	Home duties	97	12.5
	Retired/Age pensioner	60	7.8
	Other	196	25.4
**Household situation**	Single person household	65	8.4
	Living with parents	125	16.2
	Married without children	98	12.7
	Married with children	465	60.2
	Shared household, non-related	20	2.6

### 3.2 Awareness and attitude toward food waste

**Figure 1:**
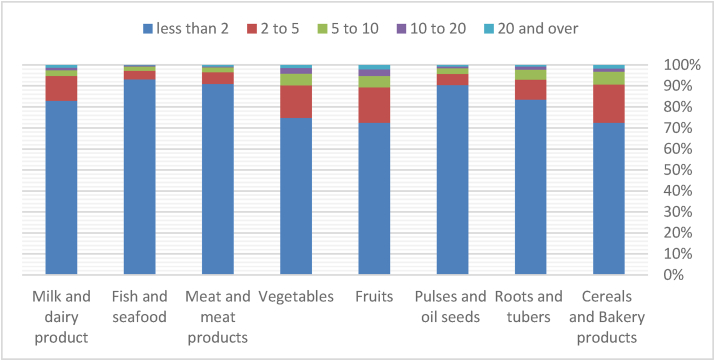
Household food waste estimation by product category (percentage).

According to the data in Figure 1, the average Iranian food waste is minimal across all food categories. Less than 2% of the food bought by Iranian families is wasted. Furthermore, cereals and baked products, fruits, and vegetables were the most often thrown away food groups. Fish and seafood, along with grains and oilseeds and meat and meat products, were the most minor wasted food groups.

As demonstrated in Table 2, when it comes to shopping habits, most respondents (60.9 %) shop at hypermarkets, and there is no significant difference across socio-demographic groups. Only 10% of those polled bought their groceries from local stores. In addition, 34.3% of the respondents were shopping once a week, and 60% were shopping once or twice a week. According to the Chi-square test, the gender variable substantially influences shopping frequency. Furthermore, 44.9% spent more than ten million IRR (approximately $50) each month on food, which is equivalent to half of Iran's minimum salary. However, the results reveal significant associations between the respondents' age and education level and monthly food expenditure (chi-square test, p < 0.05). Income and educational attainment are significant in determining food expenditure. Low education level is associated with low income and low food expenditure and vice versa. Also, 61.3 % had a shopping list. The household composition has a considerable impact on utilising the shopping list. Moreover, special deals attracted 33% of all groups.

**Table 2:** Shopping behaviour (n = 773).

Variables	Frequency	Percentage	Gender		Age		Education		Occupation		Household composition	
			χ^2^	*p*-value	χ^2^	*p*-value	χ^2^	*p*-value	χ^2^	*p*-value	χ^2^	*p*-value
**Market place**			0.870	0.649	4.950	0.764	5.170	0.273	16.000	0.091	6.180	0.631
Hypermarket	471	**60.9**										
Stores	222	28.7										
Bazar	80	10.3										
**Shopping frequency**			**16.950∗∗**	**0.002**	19.825	0.228	7.456	0.488	17.792	0.601	16.316	0.431
Every day	56	7.2										
Once every two days	115	14.9										
Twice a week	199	25.7										
Once a week	265	**34.3**										
Every two weeks	138	17.9										
**Household monthly food expenditure**			8.101	0.088	**48.766∗∗**	**0.000**	**25.747∗∗**	**0.001**	24.728	0.212	**75.492∗∗**	**0.000**
Up to 300	41	5.3										
300–500	82	10.6										
500–700	140	18.1										
700–1000	163	21.1										
More than 1000	347	**44.9**										
**Use of shopping list**			2.850	0.241	10.560	0.228	4.870	0.300	11.870	0.293	**58.480∗∗**	**0.000**
Yes	474	**61.3**										
No	102	13.2										
Sometimes	197	25.5										
**Attraction by special offers**			5.524	0.063	12.965	0.113	2.746	0.601	15.464	0.116	11.673	0.166
Yes	262	33.9										
No	253	32.7										
Sometimes	258	33.4										

∗∗*p* < 0.01, ∗*p* < 0.05.

Regarding the attitude toward food waste (Table 3), the results show that 86% of the respondents are worried about food waste and throw away very little uneaten food (65%). Also, 75% of Iranians throw away uneaten food once a week or less. In this respect, 52.4 % throw out uneaten food less than once a week, and 22.6 % never throw out uneaten food, indicating a strong commitment to responsible consumption.

**Table 3:** Attitude toward food wastage (n = 773).

Respondents	Frequency	Percentage	Sex		Age		Education		Occupation		Household composition	
			χ^2^	*p*-value	χ^2^	*p*-value	χ^2^	*p*-value	χ^2^	*p*-value	χ^2^	*p*-value
**Self-description**			3.66	0.302	**41.490∗∗**	**0.000**	4.130	0.651	**35.39∗∗**	**0.002**	**35.08∗∗**	**0.000**
I worry about food waste, and I try to avoid it	665	**86.0**										
I am aware of food waste problems, but I do not think I will change my behaviour in the near future	58	7.5										
I was interested in the issue of food waste in the past, but not anymore	16	2.1										
I do not consider food waste as a crucial problem	34	4.4										
**Throwing away uneaten food**			**11.467∗**	**0.022**	**30.857∗**	**0.014**	10.21	0.250	**36.78∗**	**0.012**	19.20	0.258
Much more than it should	3	0.4										
More than it should	14	1.8										
A reasonable amount	78	10.1										
Very little	506	**65.5**										
Almost nothing	172	22.3										
**Frequency of throwing away leftovers**			2.70	0.439	20.82	0.053	1.52	0.941	**34.00∗∗**	**0.003**	17.22	0.141
Never	175	22.6										
Less than one time a week	405	**52.4**										
From 1 to 2 times a week	139	18.0										
More than twice a week	54	7.0										
**Cooking a main meal from raw main ingredients**			3.290	0.510	18.960	0.271	**30.38∗∗**	**0.000**	**33.64∗**	**0.029**	15.00	0.525
Never	26	3.4										
Less than twice a week	79	10.2										
Three to six times	308	**39.8**										
Seven to ten times	210	27.2										
More than ten times	150	19.4										
**Eating a meal leftover from a previous day**			6.45	0.168	**27.77∗**	**0.034**	9.73	0.284	19.47	0.491	**71.23∗∗**	**0.000**
Never	57	7.4										
Less than twice a week	471	60.9										
Three to six times	226	29.2										
Seven to ten times	14	1.8										
More than ten times	5	0.6										
**Eating at the restaurant**			4.60	0.331	**39.53∗∗**	**0.001**	**27.54∗∗**	**0.001**	**37.30∗**	**0.011**	23.93	0.091
Never	283	36.6										
Less than twice a week	458	59.2										
Three to six times	25	3.2										
Seven to ten times	5	0.6										
More than ten times	2	0.3										
**Using ready-made meals**			8.30	0.081	22.93	0.116	**15.73∗**	**0.046**	**49.20∗∗**	**0.000**	**46.190∗∗**	**0.000**
Never	393	50.8										
Less than twice a week	351	45.4										
Three to six times	24	3.1										
Seven to ten times	3	0.4										
More than ten times	2	0.3										

Table 4 shows that 39.8% of respondents cook a main meal from scratch three to six times a week, while 60.9% of householders consume leftovers less than twice a week. Also, 59.2% dine out less than twice a week, and 36.6% never do, indicating that most people cook at home. Indeed, more than 90% of respondents say they use ready-made meals fewer than twice a week or never. The Chi-square test shows a significant association between self-description and age, occupation, household composition, and the behaviour of throwing away uneaten food and gender, age, and occupation.

**Table 4:** Association between labelling knowledge and socio-demographics (n = 773).

Variable	Frequency	percentage	Gender		Age		Education		Occupation		Household composition	
			χ^2^	*p*-value	χ^2^	*p*-value	χ^2^	*p*-value	χ^2^	*p*-value	χ^2^	*p*-value
**Knowledge of labelling**			**9.180∗∗**	**0.002**	**9.180∗∗**	**0.002**	3.630	0.453	2.029	0.363	**15.135∗**	**0.010**
No	226	29.2										
Yes	547	**70.8**										

∗∗*p* < 0.01, ∗*p* < 0.05.

The frequency of throwing away uneaten food also has a significant association with occupation. Furthermore, cooking the main meal from raw ingredients significantly relates to education and occupation. Meanwhile, eating a meal leftover from the previous day, which is factored in attitude variables, significantly relates to age and household composition. Eating at a restaurant significantly affects age, education, and occupation. At the same time, ready-made meal usage is significantly associated with education, occupation, and household composition.

Regarding knowledge of expiry time and labelling (Table 4), the results show that 70.8% of the respondents were knowledgeable. Statistical analysis also revealed a significant association between labelling knowledge and gender, occupation, and household composition. Regarding the amount and economic value of food wastage, the results show that 74.3% of respondents do not throw away consumable food (Table 5). Meanwhile, the economic value of food waste per month was less than 500 thousand IRR for 85.4% of respondents. The chi-square test shows a significant association between the economic value of food waste per month and respondents’ occupation (p < 0.05).

**Table 5:** The amount and economic value of food waste (n = 773).

Respondents	Frequency	Percentage	Gender		Age		Education		Occupation		Household composition	
			χ^2^	*p*-value	χ^2^	*p*-value	χ^2^	*p*-value	χ^2^	*p*-value	χ^2^	*p*-value
**Consumable food thrown away per week**			10.56	0.061	20.170	0.447	13.340	0.205	36.790	0.060	22.610	0.308
I do not throw away food	574	74.3										
Less than 250 gr	140	18.1										
Between 250 and 500 gr	40	5.2										
Between 500 gr and 1 kg	14	1.8										
Between 1 kg and 2 kg	1	0.1										
More than 2 kg	4	0.5										
**Economic value of food waste per month (10000IRR)**			0.914	0.822	8.340	0.758	6.270	0.393	29.30∗	0.015	7.160	0.846
Less than 50	660	85.4										
50–200	93	12.0										
200–500	15	1.9										
500 and More than 500	5	0.6										

∗∗*p* < 0.01, ∗*p* < 0.05.

According to the findings of what respondents do with uneaten food, 64% utilise it as animal feed, and 38% toss it away (Table 6). The most common causes for throwing food out include storing food in the fridge for an extended period of time (59.1 %), expired food (47.6 %), and food contaminated with mould (43.2%). Regarding willingness and information required to minimise food waste, respondents most often requested information on the negative effect of food waste on the environment (70.2%). Regarding the knowledge required to prevent food waste, 77.9% of respondents stated food conservation recommendations (Table 6).

**Table 6:** Behaviour regarding food waste (n = 773).

Item	Responses		Percent of Cases
	n	Percent	
**Use of uneaten food**			
I throw it in the garbage bin	294	23.8	**38.0**
I give it as a donation	111	9.0	**14.4**
I do compost	89	7.2	**11.5**
I feed it to animals	500	40.6	**64.7**
Other	239	19.4	**30.9**
**Total**	1233	100.0	159.5
**Causes of household food wastage**			
Food is expired	368	20.5	**47.6**
Food does not look good	58	3.2	**7.5**
Food has mould	334	18.6	**43.2**
Food does not have a good smell or taste	122	6.8	**15.8**
Labelling generates confusion	69	3.9	**8.9**
Food is left in the fridge for too long time	457	25.5	**59.1**
There was an error in meal planning/purchasing	7	0.4	**0.9**
Packaging was not the proper size	60	3.3	**7.8**
Poor cooking skills	37	2.1	**4.8**
Wrong preservation	85	4.7	**11.0**
Leftovers	137	7.6	**17.7**
Portions at home are too abundant	20	1.1	**2.6**
I did not like the food or ingredients	38	2.1	**4.9**
**Total**	1792	100.0	231.8
**Willingness to reduce food waste (**you would waste less food if…)			
You were better informed about the negative impacts of food waste on the environment	543	31.6	**70.2**
You were better informed of the negative impacts of food waste on the economy	444	25.8	**57.4**
The packaging of your food was more suitable	360	20.9	**46.6**
Labels were clearer	196	11.4	**25.4**
You had to pay higher taxes based on what you throw away	178	10.3	**23.0**
**Total**	1721	100.0	222.6
**Information needs to reduce food waste**			
Recipes with leftovers	315	23.3	**40.8**
Tips on how to conserve food properly	602	44.6	**77.9**
Information on the freshness of products	433	32.1	**56.0**
**Total**	1350	100.0	174.6

The clustering analysis to classify the respondents yields two different clusters (Table 7). The first cluster encompasses respondents (n = 482) (62.4%) who shop food once a week from hypermarkets/supermarkets, use the shopping list, and do not throw away any consumable food. Respondents in this cluster cook a main meal from raw ingredients three to six times a week, and they never use ready-made meals.

**Table 7:** Cluster analysis.

	**Cluster 1** (n = 482)	**Cluster 2** (n = 291)
Market place	Hypermarkets/supermarkets	Stores
Shopping frequency	Once a week	Once every two days
Use of shopping list	Yes	No
Self-description	I worry about food waste, and I try to avoid it	I worry about food waste, and I try to avoid it
Throwing away uneaten food	Very little	Very little
Frequency of throwing away leftovers	Less than one time a week	Less than once a week
Cooking a main meal from raw main ingredients	Three to six times a week	Seven to ten times a week
Eating a meal leftover from a previous day	Less than twice a week	Less than twice a week
Eating at the restaurant	Less than twice a week	Less than twice a week
Using ready-made meals	Never	Less than twice a week
Consumable food thrown away per week	I do not throw away	Less than 250 gr
The economic value of food waste per month (1000T)	Less than 50	Less than 50
Frequency (%)	62.4	37.6

The second cluster includes respondents (n = 291) (37.6%) who shop for their food from stores once every two days without using a shopping list. This group cooks a main meal from raw ingredients seven to ten times a week, uses ready-made meals less than twice a week, and throws away less than 250 g of consumable food per week. Nevertheless, both clusters are worried about food waste, avoid it whenever possible, and throw away leftovers less than once a week. However, both cluster members have a high conservation attitude and behaviour toward food waste.

### 3.3 Awareness and attitude toward food waste during COVID-19 lockdown and Ramadan

**Figure 2:**
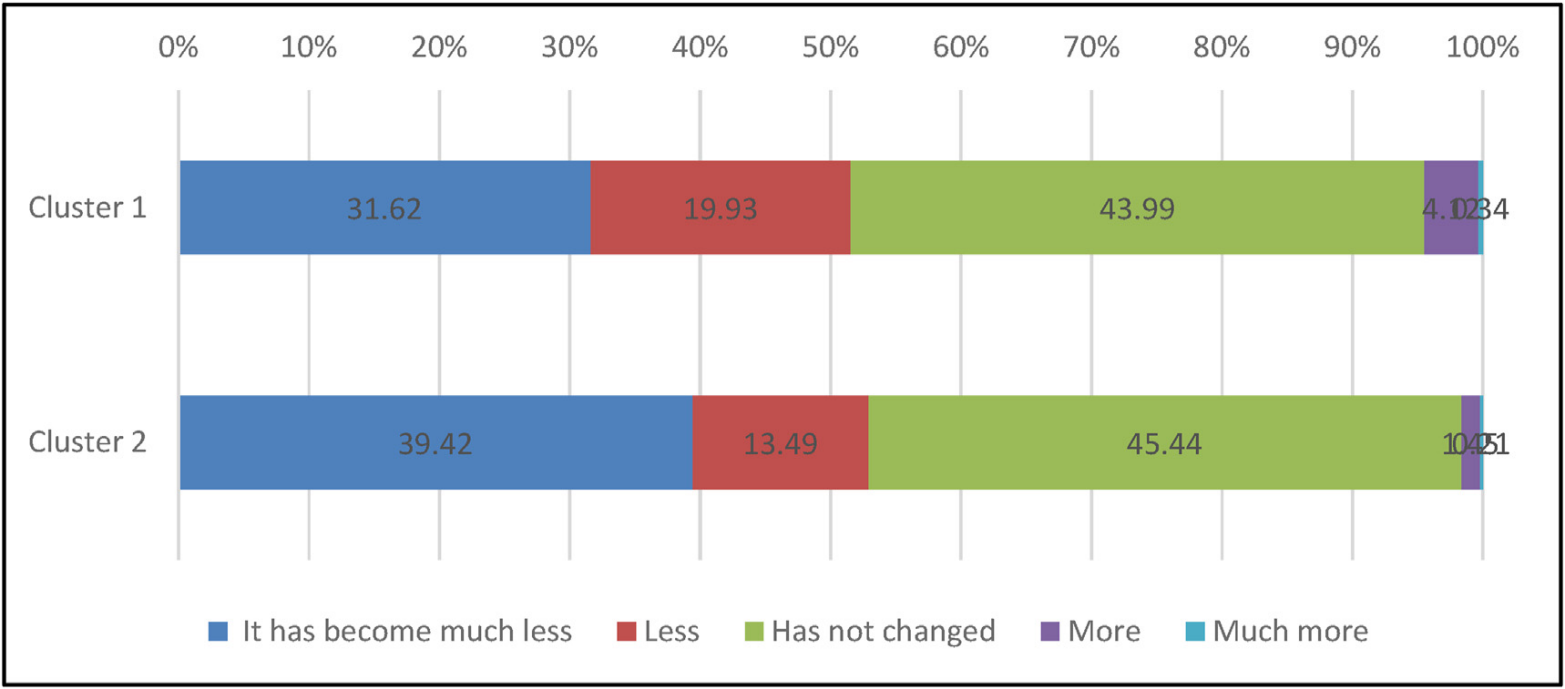
Change of food waste during COVID-19 lockdown.

**Figure 3:**
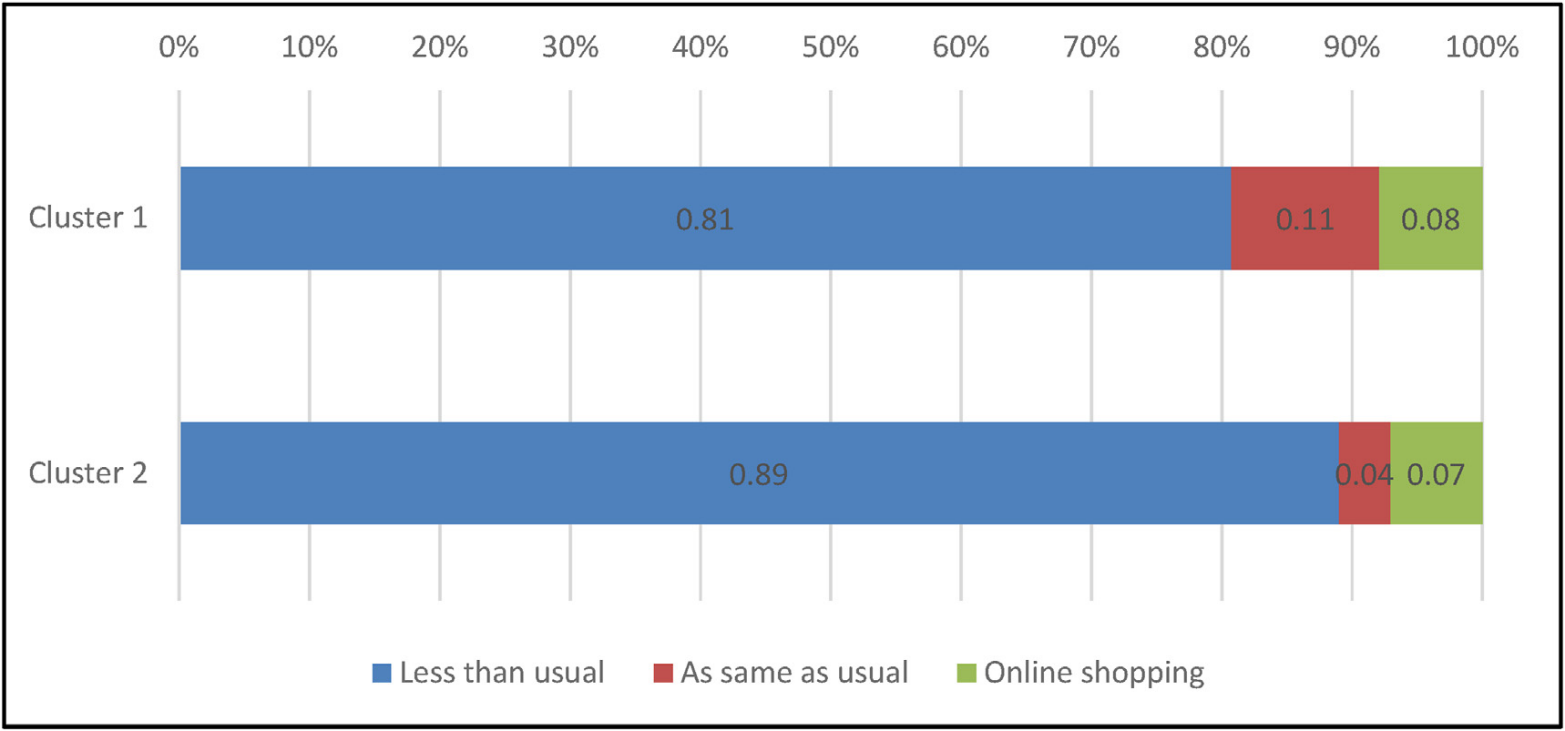
Shopping behavior during COVID-19 lockdown.

Statistical cross-analysis of the association between clusters and food waste and shopping behaviour during COVID-19 lockdown and Ramadan (Table 9) shows a significant association in 99% between the change of food waste and shopping behaviour during COVID-19 lockdown and clusters. Figures 2 and 3 show apparent differences between clusters concerning each variable (viz. food shopping behaviour, food wastage). Indeed, the chi-square p-value for change of food waste during COVID-19 lockdown was 13.25, and that for shopping behaviour and clusters was 16.38.

Table 8 revealed differences regarding food waste and shopping behaviours during COVID-19 lockdown and Ramadan. Indeed, 59.2% of the respondents have not changed their food waste behaviour during Ramadan. Meanwhile, for 52.39% of the respondents, food waste is less or much less during the COVID-19 lockdown. For only 1.94% of the respondents, food waste increased during the COVID-19 lockdown (including much and much more). Regarding the shopping amount, 70.89% of the respondents were shopping as usual during Ramadan. However, during the COVID-19 lockdown, 32.21% of the respondents increased their shopping amount (including much more and more than usual), while 41.6% kept the same amount. Regarding shopping behaviour, 55.24 % of the respondents were shopping as usual during Ramadan. However, 85.9 % of the respondents were shopping less than usual during the COVID-19 lockdown.

**Table 8:** Food wastage and shopping behaviour during COVID-19 lockdown and Ramadan.

Variables	Ramadan		Chi-square between clusters		COVID-19 lockdown		Chi-square between clusters	
	Frequency	percentage	χ^2^	*p*-value	Frequency	percentage	χ^2^	*p*-value
**Change in food waste**			7.12	0.13			13.25∗∗	0.01
Much less	218	28.20			282	36.48		
Less	82	10.61			123	15.91		
No change	458	59.25			347	44.89		
More	14	1.81			19	2.46		
Much more	1	0.13			2	0.26		
**Amount of shopping in each time**			6.31	0.18			3.08	0.54
Much more than usual	38	4.92			96	12.42		
More than usual	68	8.80			153	19.79		
As same as usual	548	70.89			322	41.66		
Less than usual	83	10.74			119	15.39		
much less than usual	36	4.66			83	10.74		
**Shopping behaviour**			2.84	0.24			16.38∗∗	0.00
Less than usual	315	40.75			664	85.90		
As same as usual	427	55.24			52	6.73		
Online shopping	31	4.01			57	7.37		

∗∗*p* < 0.01, ∗*p* < 0.05

**Table 9:** Wilcoxon test on shopping and food wastage behaviours during COVID-19 lockdown vs. Ramadan.

Variable		Mean-Rank	Z	*p-*value
Shopping behavior	COVID-19 lockdown	249.69	−14.88∗∗	0.000
	Ramadan	200.46		
Amount of shopping in each time	COVID-19 lockdown	194.08	−2.27∗	0.023
	Ramadan	201.15		
Change of food waste	COVID-19 lockdown	126.70	−6.07∗∗	0.000
	Ramadan	135.77		

The results of the Wilcoxon statistical test show that there is a significant difference regarding shopping behaviour during Ramadan and COVID-19 lockdown (z = −14.88, p < 0.01) (Table 9). Regarding the amount of shopping each time, there is also a significant difference between COVID-19 lockdown and Ramadan (z = −2.27, p < 0.05). There is also a significant difference in food waste change during Ramadan and COVID-19 lockdown (z = −6.07, p < 0.01).

**Figure 4:**
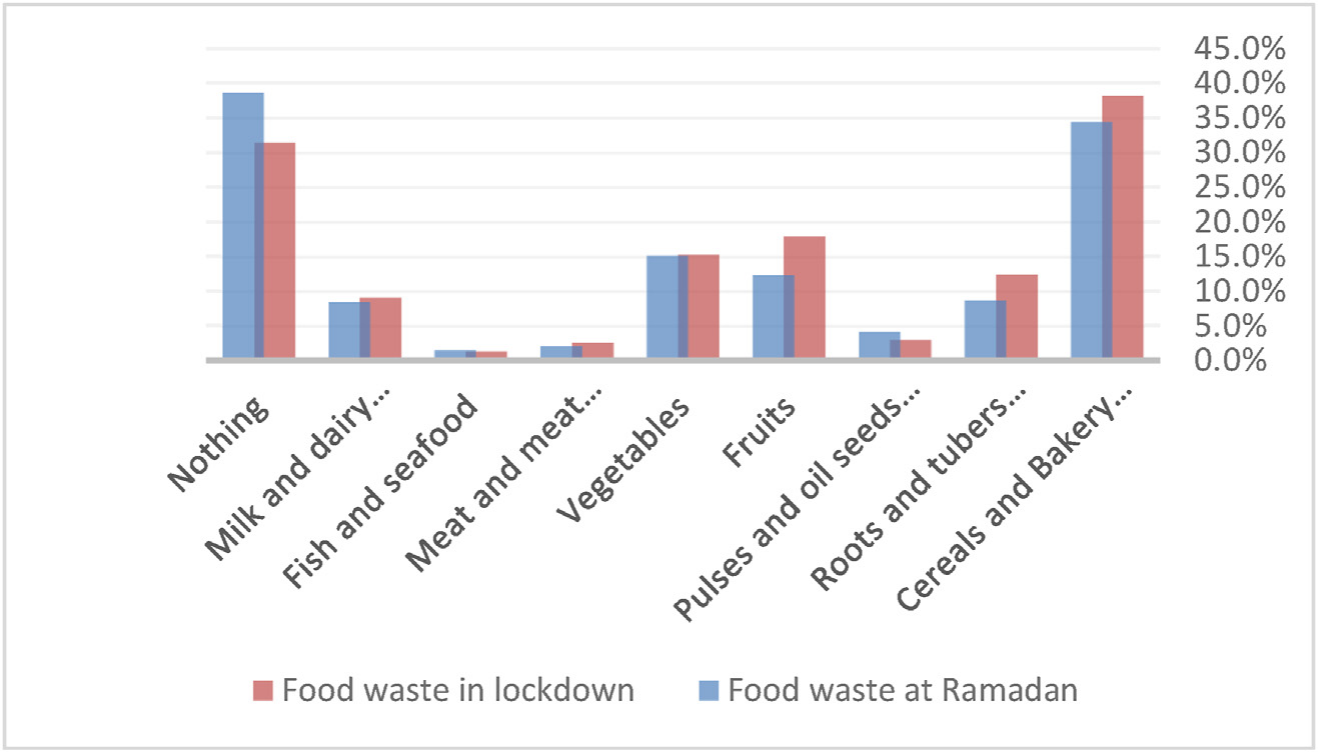
Food waste during COVID-19 lockdown vs. Ramadan.

Figure 4 shows no difference in each food group's wastage during Ramadan vs. during the COVID-19 lockdown. Indeed, the most wasted food groups during both periods are cereals and bakery products (bread, rice, pasta, etc.) followed by vegetables and fruits.

## 4 Discussion

This study aimed to examine the impact of COVID-19 on Iranian consumers' knowledge, attitudes, and behaviours about food waste. The study's findings revealed several consumer trends in Iranians' food consumption, food waste, and food purchasing behaviours during the pandemic.

First, Iran has a low rate of household food waste as well as a positive attitude toward food waste prevention and reduction. These findings align with regional averages for South and Southeast Asia (which includes Iran) (FAO, 2011). A variety of factors might be at play here. The data revealed a link between food pricing, preservability, and food waste. Indeed, as shown in various countries in the NENA region (Capone et al., 2016), such as Algeria (Ali Arous et al., 2017), Egypt (Elmenofi et al., 2015), Lebanon (Charbel et al., 2016), Morocco (Abouabdillah et al., 2015), Tunisia (Sassi et al., 2016) and Turkey (Yildirim et al., 2016), cereals and baked products, fruits, and vegetables were the food items that were most often thrown out. The increased waste of bakery items may be related to the large amount of products purchased and utilised by Iranian households (Tootyaii and Soleimani, 2009).

Furthermore, to ensure societal access to basic foodstuffs, the government subsidises the majority of bakery items and so has cheap costs. There is a greater possibility that they will be thrown away. For instance, bread is both the most essential source of nourishment and a key element in Iranian cuisine (Karizaki, 2017; Pontonio et al., 2015). As a result of government subsidies, it has long been the most affordable staple food in Iran (Radio Farda, 2019). Because of this, Iranians are second only to Turkish in terms of bread consumption per capita worldwide, and their consumption is over double the average in European nations (Jafari et al., 2016). In July 2021, Iranians were purchasing less bread since the government hiked prices for subsidised flour. Indeed, two weeks after prices rose 12% to as high as 30% in certain areas, industry sources claim consumers are purchasing 20–30% less bread, despite being an essential food item for wage workers (Iran International, 2021). Despite their high cost, food subsidies remain politically risky to abolish or even reform. The 2010 targeted subsidies reform Act aimed to replace food subsidies with cash transfers progressively. Nonetheless, the follow-up phase of the reforms was postponed by Parliament in 2012, subsidised product consumption rebounded, and the overall reform remains stalled (Michel, 2019).

On the other hand, fruits and vegetables are especially perishable items that may end up in the bin if they are not adequately conserved. In contrast, fish and seafood, along with grains and oilseeds, and meat products, were the food groups with the least amount of food wasted. While the low waste of fish and seafood may be owing to their high pricing, the low waste of pulses and oilseeds may be due to their excellent preservability. In reality, recent increases in food price inflation have influenced the food basket composition of ordinary Iranian families. According to Najaf Abadi Kazemi et al. (2020), red meat intake per capita declined to 6 kg per year in 2016/17, decreasing from 8.7 kg in 2011/12. Since 2016/17, food costs have increased by 120%, with vegetables seeing the most significant increase at 163%. In addition, higher import costs and trade restrictions will keep inflation over 20% in the following years (World Bank, 2020b).

Second, the findings indicated that most respondents cook and prepare meals at home, resulting in a reduction in the number of leftovers thrown away. The high percentage of respondents who cook might suggest that also Iranians became “resourceful planners and cooks” as Yetkin Özbük et al. (2021) defined them in Turkey. The pandemic strengthened this trend as many restaurants and food services were closed during the lockdown, so people were cooking more and spending more time in the kitchen compared to the pre-lockdown period (Kumar and Dwivedi, 2020). Cooking skills generally help increase flexibility to respond to occasional food shortages (Bender et al., 2021) and decrease food wastage (Rodgers et al., 2021b). Indeed, Sharp et al. (2021) found that lower levels of food waste correlated with higher cooking confidence levels. It is uncommon to eat outdoors or to purchase ready-made cuisine in Iran. As a result, most responders have devised ways to preserve, store, and consume leftovers. Financial reasons might explain this. Eating out is often more costly than cooking and eating at home. Iranian consumers are paying greater attention to their budgets and avoiding superfluous spending due to the country's limited economic development and high inflation. The findings demonstrated that positive consumer attitudes toward food waste avoidance are more likely to be influenced by financial issues (such as inflation and the economic crisis) than by a pro-environmental concern. Meanwhile, information on the negative effect of food waste on the environment is the most often requested information by those who answered the survey questions.

Third, regarding shopping habits, the results revealed that most respondents shopped at hypermarkets. These findings are similar to those obtained in other NENA countries such as Algeria (Ali Arous et al., 2017), Lebanon (Charbel et al., 2016), Morocco (Abouabdillah et al., 2015) and Tunisia (Sassi et al., 2016), while results were different in Egypt (Elmenofi et al., 2015). The survey findings confirm that Iranian society is moving toward a more contemporary urban lifestyle. In fact, the country has lately seen changes in the retail food procurement sector, known as the “supermarket transition,” as shown by the emergence of hypermarkets and supermarkets, as has been observed in other countries in the NENA region (Seyfert et al., 2014). Moreover, the results highlighted that most respondents used a shopping list, explaining the low food waste. In fact, shopping behaviour is considered key to reducing food waste (UNEP, 2014). Shopping became a more ‘rational and planned activity’ during the pandemic since people went shopping less frequently than before – as shown in many countries such as Bosnia and Herzegovina (Ben Hassen et al., 2021d), Serbia (Berjan et al., 2021), or Turkey (Songur Bozdag and Çakiroglu, 2021) – so they had to know in advance what they need, and the use of a shopping list became more common than before the pandemic. However, Vidal-Mones et al. (2021) highlighted that buying more food than usual due to anxiety or fear and improvising when buying groceries affected household food wastage in Spain. Further, the cluster analysis reveals that both clusters are worried about food waste, avoid it whenever possible, and throw away leftovers less than once a week.

Furthermore, regarding the immediate impacts of COVID-19 on consumer attitudes and behaviours, the findings revealed substantial changes in how consumers buy and interact with food. In the first place, the study revealed that food waste decreased during the COVID-19 pandemic. Because restaurants and coffee shops are closed, most meals are prepared and enjoyed at home, resulting in fewer leftovers being thrown away. Different studies have shown a similar decrease in food wastage during the pandemic in countries such as Italy (Amicarelli et al., 2021a, Amicarelli et al., 2021b; Pappalardo et al., 2020; Vittuari et al., 2021), Lebanon (Ben Hassen et al., 2021c), Colombia (Mejia et al., 2021), Qatar (Ben Hassen et al., 2020), Bosnia and Herzegovina (Ben Hassen et al., 2021d), Serbia (Berjan et al., 2021) as well as in the European Union (Bel and Marengo, 2021). In general, reducing food wastage requires improving purchase planning, knowledge of labels, and food storage and cooking skills (Vásquez Neyra et al., 2022).

Second, as observed in many countries such as Lebanon (Ben Hassen et al., 2021c), Oman (Ben Hassen et al., 2022a), Russia (Ben Hassen et al., 2021a), Serbia (Ben Hassen et al., 2021d), and Bosnia (Ben Hassen et al., 2021d) shoppers decreased the number of shopping visits they made and shopped less often, decreasing their perceived risk of COVID-19 exposure by purchasing more items on each trip, lowering their perceived risk of COVID-19 exposure.

Interestingly, the findings indicated that, contrary to what other studies have shown in the NENA area, where food waste rises during the fasting month of Ramadan (Abiad and Meho, 2018; Baig et al., 2019; FAO, 2014), the results revealed that during that month, food waste did not increase in Iran. Typically, Ramadan is marked by the preparation of meals that surpass the need of families. However, due to the economic crisis, Iranian consumers pay close attention to their spending habits. As a matter of fact, Iranian economic activity and government income are mainly based on oil and gas exports, and as a result, the country remains subject to the considerable volatility of oil prices (World Bank, 2020a). The restoration of US sanctions against Iran in May 2018 had a devastating impact on the nation's economy, with the amount of oil exported by the country dropping significantly. Consequently, Iran had a significant decline in oil output, resulting in a significant income loss. As a result, the oil industry fell by 14.1% in 2018/19. Construction, basic metals, and petrochemicals are just a few of the key non-oil industries that have seen sanctions increases in recent months. As a result, Iran has seen two years of severe economic decline, with the gross domestic product (GDP) contracting by 4.7% in 2018/19 and 8.2% in 2019/20. As a result of negative economic growth and excessive inflation, household livelihoods are further threatened, with a loss of buying power and a rise in poverty (Najaf Abadi Kazemi et al., 2020).

## 5 Conclusion

Reducing food losses and waste is paramount to ensure sustainable access to food in a country such as Iran, suffering from an economic recession due to US sanctions - with a decrease in purchasing power. This is even more important in the context of the pandemic that determined far-reaching impacts on the whole food chain. In particular, the survey results show that the pandemic affected Iranian households' purchasing habits and consumption behaviours. Changes were particularly evident during the lockdown period due to movement restrictions and some shops' closing (including restaurants and catering services). While the COVID-19 pandemic represents a great challenge for policymakers, the disruptions that it has brought about in food-related habits and behaviours can also represent a ‘window of opportunity’ to move towards more sustainable consumption patterns by, among others, curbing the amount of household food wastage, as observed in several countries (Nemes et al., 2021). Therefore, recovery strategies should consider these changes instead of emergency plans to revisit food-related policies (including access to food and food security). In this respect, it is of paramount importance to combine informational, economic, and legal policy instruments to reduce food waste in Iran. As for informational instruments, the survey's insights show that the focus of awareness-raising campaigns should be on economics rather than on environmental aspects to yield the best results in Iran. However, as Stöckli et al. (2018) highlighted, conceptual and empirical evidence indicates that informational interventions are relatively ineffective. Consequently, we suggest that non-informational intervention types, such as prompts, and rewards, should be considered. Also, the cultural background should be exploited in awareness campaigns.

However, there are certain limitations to the survey technique and tool that limit the sample representativeness. First, the sample bias is the key limitation of this study. Participants in the survey were selected at random, hired voluntarily, and not compensated. Consequently, the questionnaire was self-administered, and completed only by individuals motivated by an interest in the subject. As a result, our survey does not reflect the general Iranian population. Individuals with a high level of education, for example, were overrepresented in our sample. Low-education people are often underrepresented in surveys (Spitzer, 2020). In addition, people who cannot use the internet and the elderly are often left out of online polls. Low-income families and informal workers in the NENA area, in particular, had the lowest participation rates in online surveys. They may not participate in online surveys because of inadequate technology, digital illiteracy, or a lack of incentives (Atamanov et al., 2020). The limitations described above are typical in Computer Assisted Web Interviewing (CAWI), which is commonly used in surveys (Couper, 2000; Evans and Mathur, 2018; Monzon and Bayart, 2018). Because of this bias, it is not easy to generalise the survey results to the whole Iranian population. However, because of the COVID-19 pandemic, online studies may gather data from a distance, which is an obvious benefit when social distance is necessary and face-to-face research is challenging.

Second, our measurement of food waste was self-reported and point-in-time. However, immediacy bias could affect how people perceive their food waste reduction during the lockdown and Ramadan. Furthermore, as Rodgers et al., 2021a, Rodgers et al., 2021b highlighted, changes in food-related habits were confounded by conformity with general health guidelines in light of the pandemic, which could thus have mirrored current societal expectations during the pandemic's early months. Also, since individuals are conscious of and sensitive to societal expectations surrounding food waste (Stancu et al., 2016), this could have affected this research's results.

To the best of our knowledge, this is the first research on people's views of the effects of COVID-19 on food waste in Iran and the NENA area. Future research should investigate whether and to what degree customers can uphold the virtuous anti-waste food management practices acquired during the lockdown. Because the COVID-19 pandemic is a recent phenomenon with an indefinite duration, more data and information are required to assess its impact on food consumption. This study and others in the future will help the government prepare for future disasters and pandemics. Although the current research focuses on the immediate and short-term consequences of the pandemic, additional studies are needed to understand better its long-term effects on food-related behaviour patterns (e.g. food shopping/sourcing, consumption, preparation) and on food and nutrition security in the country. It may be possible to examine how innovations and coping mechanisms adopted by consumers help or hinder the transition to more sustainable food and consumption systems during global crises like the COVID-19 pandemic (Nemes et al., 2021).

## Declarations

### Author contribution statement

Mohammad Sadegh Allahyari, Soroush Marzban: Conceived and designed the experiments; Performed the experiments; Contributed reagents, materials, analysis tools or data; Wrote the paper.

Hamid El Bilali, Tarek Ben Hassen: Analyzed and interpreted the data; Wrote the paper.

### Funding statement

This work was supported by Qatar National Library.

### Data availability statement

Data will be made available on request.

### Declaration of interest’s statement

The authors declare no conflict of interest.

### Additional information

No additional information is available for this paper.
